# Experimental and Theoretical Investigation of the *κ*-Phase (Ag_2_Mg_5_) in the Ag-Mg System

**DOI:** 10.3390/ma19112241

**Published:** 2026-05-26

**Authors:** Weronika Gozdur, Wojciech Gierlotka, Magdalena Bieda, Władysław Gąsior, Andrzej Budziak, Marek Polański, Magda Pęska, Adam Dębski

**Affiliations:** 1 Institute of Metallurgy and Materials Science, Polish Academy of Sciences, 25 Reymonta St., 30-059 Krakow, Poland; w.gozdur@imim.pl (W.G.); m.bieda@imim.pl (M.B.); w.gasior@imim.pl (W.G.); 2 Department of Materials Science and Engineering, National Dong Hwa University, Hualien 974301, Taiwan; wojtek@gms.ndhu.edu.tw; 3 Faculty of Energy and Fuels, AGH University of Krakow, Al. Mickiewicza 30, 30-059 Krakow, Poland; budziak@agh.edu.pl; 4 Department of Functional Materials and Hydrogen Technology, Military University of Technology, 2 Kaliskiego St., 00-908 Warsaw, Poland; marek.polanski@wat.edu.pl (M.P.); magda.peska@wat.edu.pl (M.P.)

**Keywords:** *κ*-Ag_2_Mg_5_ phase, enthalpy of formation, thermodynamic properties, calorimetry, ab initio calculation, defect formation energy

## Abstract

The present study extends the investigation of thermodynamic properties of phases in the silver–magnesium binary system, with particular emphasis on the *κ*-Ag_2_Mg_5_ phase, for which available literature data remain scarce. The work is divided into two parts. The experimental section comprises the synthesis of the *κ* phase from high-purity Ag and Mg, followed by its characterisation using scanning electron microscopy (SEM) and X-ray diffraction (XRD). The synthesised material was subsequently used for calorimetric determination of the standard enthalpy of formation employing the drop solution method. Measurements were carried out in two experimental series (A and B), using two different metallic solvents (Al and Sn), at temperatures of 1020 K and 689 K. The enthalpy of formation obtained in both series was −14.4 ± 0.32 and −14.5 ± 0.42 kJ/mol at., respectively. In addition, the limiting partial enthalpy of solution of liquid Ag in liquid Al was determined calorimetrically and its average value is equal 7.1 ± 0.7 kJ/mol. The theoretical part of the study involved ab initio calculations of defect formation energies. The obtained results show good agreement with available literature data and provide a consistent interpretation of the observed non-stoichiometry of the *κ*-phase.

## 1. Introduction

The scope of applications of Ag-Mg alloys is diverse, ranging from advanced materials through energy technologies and extending to medical industry applications [1]. Understanding existing intermetallic compounds, their thermal stability limits, and invariant reactions is important for shaping the material’s microstructure intentionally [2]. For instance, in the aerospace industry, magnesium alloys containing silver are valuable due to their strength-to-weight ratio. Understanding phase equilibria enables the optimisation of the casting and heat treatment process, resulting in enhanced creep resistance [3,4]. At the same time, Ag-Mg alloys are considered modern biomaterials for implants such as bone screws, plates and pins. This is connected with the similar density and Young’s modulus of the Mg-based alloys and bone. The addition of silver to Mg-based alloys leads to improvement of the mechanical properties and enhances antibacterial properties through the release of the Ag ions [5,6,7,8]. Additionally, magnesium exhibits good degradation characteristics, thanks to which corrosion of alloys would eventually result in the complete degradation of the implant in the body [8,9,10]. Furthermore, due to the relatively high gravimetric capacity of magnesium hydride (7.6 mass %), it is considered a hydrogen storage material. However, it should be noted that the practical application of this material is limited by slow ab-/desorption and high temperature of the reaction [8,9]. Research indicates that modifying Mg-based alloys by alloying additives (such as Cu, Pd, Al, Ni, Ag) may slightly improve the kinetics of hydrogen sorption [11,12,13,14]. Lastly, it should be highlighted that due to the optical and emissive properties of Ag-Mg-based alloys, they are potential candidates to be utilised in the field of electronics. Selected phases from the system can be used as staple electrodes for organic light-emitting diodes (OLEDs) [15].

The phase diagram of the Ag-Mg system is characterised by considerable complexity, particularly on the Mg-rich side [16,17]. Recent literature indicates the presence of solid solutions based on (Ag) of the FCC structure and (Mg) of the HCP structure and, intermediate phases such as Ag_3_Mg and the congruently melted AgMg phase [18,19]. As the magnesium content increases above 50% more complex phases are observed including *ε*′ (Ag_17_Mg_54_), *ε* (AgMg_3_) and polymorphic *γ* and *γ*′ (AgMg_4_) varieties [16,17]. An important aspect of the modern redefinition of the Mg-rich part of the system was the identification of the low-temperature *κ*-Ag_2_Mg_5_ phase characterised in the Kudla doctoral thesis [16]. This work makes a significant contribution to the thermodynamic description of the system. The observation of the *κ* phase was later confirmed in the work of Castro et al. [20]. This intermetallic compound crystallises in a hexagonal system (space group: P6_3_/mmc, Pearson symbol: hP28) and adapts the prototypical structure Co_2_Al_5_ [20]. The upper stability limit of the phase determined to be 440 °C, and it is formed as a result of the peritectoid reaction according to the following reaction: AgMg + *ε*-AgMg_3_ ↔ *κ*-Ag_2_Mg_5_ [16]. In our previous work [17], thermodynamic properties, equilibrium system calculations and detailed descriptions of historical research on the Ag-Mg system were presented. The re-calculated system took into account the occurrence of the *κ* phase and additionally, thanks to the use of the ab initio calculations, the formation energy of the *κ*-phase at 0 K was determined. Furthermore, computational methods enable the identification of selected physical and mechanical properties such as elastic modulus, hardness and heat capacity of the *κ*-Ag_2_Mg_5_ [17].

As it has been outlined, there is a limited number of works on the *κ* phase in the currently available literature. The thermodynamic properties of the phase are mostly based on calculations. In view of the above, the present study serves as a continuation of the research [17,21] on the thermodynamic properties of the Ag-Mg system. The main objective of this work is to expand current knowledge, especially on the *κ*-Ag_2_Mg_5_ phase. The scope of work has been divided into two parts. The experimental part of the study includes the synthesis of *κ*-phase obtained from high-purity Ag and Mg, as well as the results of the analysis from the scanning electron microscopy (SEM) and X-ray diffraction (XRD). Prepared material was then utilised for calorimetric measurements of the standard enthalpy of formation with the use of the drop solution calorimetry method. In the theoretical part of the study, defect formation energy was determined using the ab initio calculations. The obtained results demonstrate good agreement with the extant literature and provide a coherent interpretation of the observed non-stoichiometry of the *κ*-phase. 

## 2. Materials and Methods

The results presented in this work were derived based on the analyses of experimental studies in which the enthalpy of formation of *κ*-phase was determined. The second part of the study involves the theoretical investigation with the aim of an ab initio calculation of the defect formation energy in the mentioned phase. In the remainder part of the section, all the methods used are described in detail.

### 2.1. Experimental Investigation

The first stage (part) of the experimental study of this work was the sample preparation from high-purity silver and magnesium in a glove box (Labmaster, Mbraun, Garching, Germany) under a high-purity argon atmosphere (H_2_O < 0.5 ppm, O_2_ < 0.1 ppm). Weighted proper amounts of metals with the 0.1 mg precision were melted in a magnesium oxide crucible (INN-THERM, Trzcianka, Poland) in a resistance furnace. The obtained liquid alloy was carefully stirred and poured into a specially designed steel casting mould. Next, after the preparation, the obtained alloy was cut into smaller pieces, encapsulated in a quartz tube and annealed for 27 days at 360 °C in a resistance furnace (Fine Instruments, Krakow, Poland). To protect the alloy from potential reaction with the quartz ampule during the annealing process, alloy fragments were placed in a protective steel crucible. Detailed information on all materials applied is presented in Table 1.

Observations of the alloy samples before and after annealing were conducted using the Quanta 3D FEG microscope (Thermo Fisher Scientific, Eindhoven, The Netherlands) equipped with Energy-Dispersive X-ray Spectroscopy (EDS) (EDAX Amtec, Pleasanton, CA, USA). The chemical composition was determined by EDS and quantification was performed using the EDAX ZAF correction methods in standardless mode. Additionally, elemental mapping was performed.

The XRD measurements were performed for a powder sample from the alloy after annealing with the usage of a Panalytical Empyrean diffractometer with Cu-Kα radiation (λ = 1.54 Å). The analysis of diffraction patterns was performed with the HighScore version 4.8 (Malvern Panalytical, Malvern, UK) software connected to the PDF5+ database software version 25.1 (ICDD, Newtown Square, PA, USA). Additionally, a quantitative phase analysis was carried out using the Rietveld refinement method implemented in JADE software version 9.4 (MDI, Materials Data Inc., Livermore, CA, USA), using crystallographic data from the PDF-5+ database. The refinement was performed using the whole pattern fitting (WPF) approach, including refinement of scale factors, peak profile parameters (pseudo-Voigt function) and preferred orientation.

The standard enthalpy of formation was determined with the drop solution calorimetry method in two measurement series. In measurement series A, aluminium was used as the metallic solvent, while in series B, tin was utilised. The enthalpy value was calculated based on the difference in thermal effects associated with the heating of the sample from room temperature (*T*_D_) to the measurement temperature (*T*_M_), and the dissolution of the alloy and its components in the liquid metallic solvent. The final value was calculated according to Equation (1):

(1) $${∆}_{f}H\;=\;x_{\mathrm{Ag}}{∆H}_{\mathrm{Ag}}^{0}+\;x_{\mathrm{Mg}}{∆H}_{\mathrm{Mg}}^{0}-{\Delta H}_{x_{\mathrm{Ag}}x_{\mathrm{Mg}}}^{0}$$

where ${∆}_{f}H$ is the standard enthalpy of formation of investigated alloy; *x*_Ag_ and *x*_Mg_ are the mole fractions of the alloy components; and ${∆H}_{\mathrm{Ag}}^{0}$, ${∆H}_{\mathrm{Mg}}^{0}$ and ${\Delta H}_{x_{\mathrm{Ag}}x_{\mathrm{Mg}}}^{0}$ are the heat effects associated with the dissolution of one mole of the alloy components and the alloy (phase) itself in liquid solvent (Al or Sn). The ${∆H}_{\mathrm{Ag}}^{0}$ and ${∆H}_{\mathrm{Mg}}^{0}$ are calculated with the use of Equations (2) and (3).

(2) $${∆H}_{\mathrm{Ag}}^{0}=\Delta_{\mathrm{sol}}{\bar{H}}_{\mathrm{Ag}\left(l\right)}^{\infty }+{∆H}_{\mathrm{Ag}}^{T_{D}\to T_{M}}$$

(3) $${∆H}_{\mathrm{Mg}}^{0}=\Delta_{\mathrm{sol}}{\bar{H}}_{\mathrm{Mg}\left(l\right)}^{\infty }+{∆H}_{\mathrm{Mg}}^{T_{D}\to T_{M}}\;$$

These represents sum of the of the limiting partial enthalpy of solution of liquid Ag ($\Delta_{\mathrm{sol}}{\bar{H}}_{\mathrm{Ag}\left(l\right)}^{\infty }$) and Mg ($\Delta_{\mathrm{sol}}{\bar{H}}_{\mathrm{Mg}\left(l\right)}^{\infty }$) in liquid Al [22] or Sn [23,24], and the enthalpy change of the pure elements heated from room temperature to the measurement temperature (${∆H}_{\mathrm{Ag}}^{T_{D}\to T_{M}}$, ${∆H}_{\mathrm{Mg}}^{T_{D}\to T_{M}}$) calculated using relations in [25]. The measurements were performed by means of the Setaram MHTC 96 line evo calorimeter (Setaram instrumentation—KEP technologies, Caluire, France), with a procedure similar to that described in our previous work [26]. Each measurement series began with a preparation stage, during which an alumina crucible with the metallic solvent was placed in the calorimeter. Subsequently, the air was evacuated from the calorimeter using a vacuum system. After reaching the acceptable vacuum level, the device was flushed several times and filled with high-purity argon. Thereafter, the calorimeter was heated up to measurement temperature, which was 1020 K for series A and 689 K for series B. After thermal equilibration of the device, the calibration constant was determined based on the thermal effect of approximately 6 calibration samples. The calibration material was the same as the solvent material. Once the calibration was completed, small samples made from alloy with irregular shape and the average mass equal to 0.0830 (±0.009) g were dropped into the reaction crucible with the metallic bath. The thermal effects were registered and analysed with Calisto software v. 1.39 (Setaram instrumentation—KEP technologies, Caluire, France).

### 2.2. Theoretical Investigation

Our previous work on the Ag-Mg [17] system included theoretical predictions of the formation energies of intermetallic compounds, as well as the determination of their elastic properties. It is therefore worthwhile to extend this theoretical study and focus specifically on the Ag_2_Mg_5_ phase. As in the previous work, ab initio calculations based on density functional theory (DFT) were employed, using the VASP software (version 6.4.1) [27]. The calculations utilised the generalised gradient approximation (GGA) with the Perdew–Burke–Ernzerhof (PBE) parameterisation [28], along with the projector augmented wave (PAW) method [27] to describe ion–electron interactions. A cut-off energy of 350 eV and a Γ-centred k-point mesh with a density of 0.09 Å^−1^ were used; the cut-off energy was set to at least 1.3 times higher than the maximum value specified in the pseudopotential file [27]. For the calculation of electronic properties, the cut-off energy was increased to 500 eV, and a denser k-point mesh with a density of 0.05 Å^−1^ was applied. For defect formation energy calculations, a 2 × 2 × 2 supercell was used. This supercell size was sufficient to model various types of defects due to the relatively large unit cell, which contains 28 atoms (8 Ag and 20 Mg).

The defect formation energy was calculated according to the well-known Equation (4):

(4) $$E^{f}\left[X\right]\;=\;E\left[X\right]\;-{\;E}_{\mathrm{pristine}}\;-\sum_{i}n_{i}\mu_{i}$$

where $E^{f}\left[X\right]$ is formation energy of defect *X*, *E*[*X*] is energy of defected supercell, *E_pristine_* is energy of pristine (undefected) supercell, $n_{i}$ is number of atoms i taken from or given to reservoir, and $\mu_{i}$ is chemical potential of atom *i* (*i* = Ag, Mg).

Since the Ag_2_Mg_5_ phase shows metallic character, there are no charged defects, and thus the correction term in Equation (4) is omitted.

## 3. Results and Discussion

### 3.1. Experimental Investigation

#### 3.1.1. Structural Analysis and Microstructure Observation

**Figure 1:**
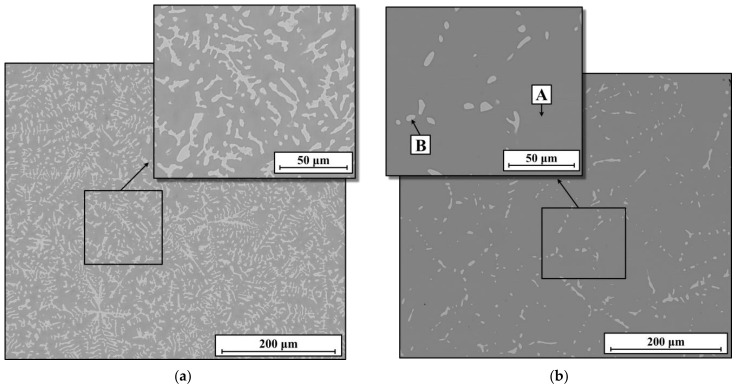
BSED/SEM images of the sample from alloy 1 (**a**) before homogenisation and (**b**) after homogenisation.

The microstructure of the alloy before and after homogenisation for 27 days at 360 °C was observed with the use of the SEM. The resulting microstructure images collected with the Backscatter Electron Detector (BSED) are presented in Figure 1a,b.

The BSED/SEM images reveal differences in the material’s microstructure. Figure 1a presents the microstructure of the as-cast alloy before annealing, which shows two phases and is clearly non-homogeneous. Phase A corresponds to the κ-Ag_2_Mg_5_ intermetallic phase, whereas phase B was identified as the Ag_1.056_Mg_0.944_ one. Annealing reduced the amount of phase B, as seen in Figure 1b. During annealing, chemical components redistribute between regions with different local concentrations, accompanied by partial dissolution of metastable phases. This process increases the volume fraction of phase κ-Ag_2_Mg_5_ and decreases that of phase Ag_1.056_Mg_0.944_, thereby driving the alloy toward a more thermodynamically stable equilibrium state. The EDS analysis revealed that the average chemical composition of the alloy remained nearly unchanged after homogenisation, indicating that the heat treatment did not alter the global Ag/Mg ratio but rather affected the local elemental distribution and phase constitution. The measured compositions before homogenisation (Ag = 26.8 ± 1.2 at. %, Mg = 73.2 ± 0.8 at. %) and after homogenisation (Ag = 27.5 ± 1.3 at. %, Mg = 72. 5 ± 0.7 at. %) are within the experimental uncertainty of the EDS method. In the as-cast condition, rapid solidification and limited atomic mobility promote microsegregation, leading to the formation of Mg-rich and Ag-enriched phases. During homogenisation, enhanced solid- state diffusion reduces chemical heterogeneity and drives the alloy toward thermodynamic equilibrium. After homogenisation, the dominant phase became the κ-Ag_2_Mg_5_ intermetallic phase, which is consistent with the average alloy composition. Simultaneously, a minor of Ag_1.056_Mg_0.944_ phase was still detected, indicating that local Ag-Enriched regions were not eliminated completely during the applied homogenisation. This suggests that the diffusion time and/or temperature were sufficient to reduce segregation but insufficient for complete thermodynamic equilibration throughout the material. Therefore, the compositional variations observed in the EDS maps are attributed primarily to residual microsegregation inherited from solidification and subsequent diffusion- driven phase redistribution during homogenisation.

The local chemical composition analysis of the sample after annealing is summarised in Table 2.

**Figure 2:**
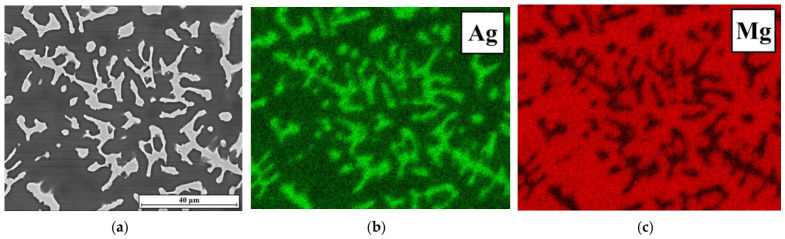
EDS maps for the alloy before annealing. (**a**) BSE image of the analysed area, (**b**) Ag distribution map, (**c**) Mg distribution map.

**Figure 3:**
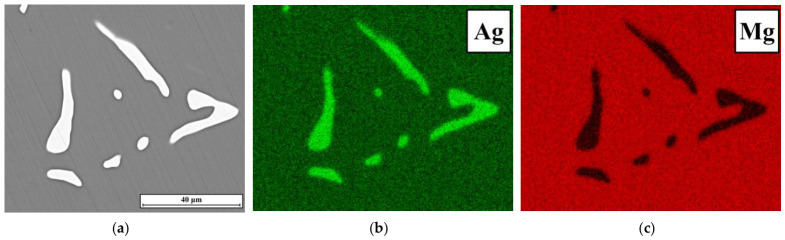
EDS maps for the alloy after annealing. (**a**) BSE image of the analysed area, (**b**) Ag distribution map, (**c**) Mg distribution map.

The EDS maps presented in Figure 2 and Figure 3 demonstrate the distribution of the elements in within the investigated microstructures before and after the annealing process. The distribution of the Ag is illustrated in green, while the distribution of Mg is shown in red.

An analysis of the obtained maps highlights a difference among the phases present in the studied material. For phase A (Figure 1b), a higher content of magnesium was observed in comparison to phase B (Figure 1b). More detailed analysis of the chemical composition of the homogenised alloy, presented in Figure 1b confirmed the difference within the composition of the two distinct phases. The results of the local analysis for both phases are summarised in Table 3. Furthermore, EDS analysis revealed no contaminants in the sample that might have been introduced during sample preparation.

**Figure 4:**
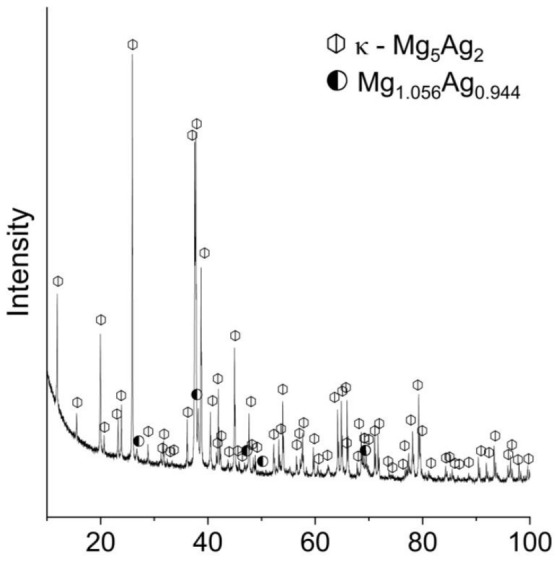
XRD diffraction pattern of powder sample from the alloy after homogenisation.

The diffraction pattern presented in Figure 4 indicates the presence of two phases in the analysed alloy, with *κ*-Ag_2_Mg_5_ as the dominant phase. Quantitative Rietveld analysis revealed that *κ*-Ag_2_Mg_5_ constitutes approximately 98 wt.%, while the remaining 2 wt.% corresponds to a minor Ag_1.056_Mg_0.944_ phase, which is close to the stoichiometric AgMg composition. The quality of the refinement was satisfactory, as indicated by a low-profile residual (Rp ≈ 6.3%) and good agreement between the experimental and calculated patterns.

This material was subsequently utilised in further studies of the standard enthalpy of formation.

#### 3.1.2. Enthalpy of Formation Measurements

The values of the limiting partial enthalpy of solution for elements ($\Delta_{\mathrm{sol}}{\bar{H}}_{\mathrm{Ag}\left(l\right)}^{\infty }$, $\Delta_{\mathrm{sol}}{\bar{H}}_{\mathrm{Mg}\left(l\right)}^{\infty }$) in liquid tin used for series B were adapted from our previous works [23,24]. In the case of the measurement series A, the value of the limiting partial enthalpy of solution for liquid Mg in liquid Al ($\Delta_{\mathrm{sol}}{\bar{H}}_{\mathrm{Mg}\left(l\right)}^{\infty }$) was similarly adapted from previous research [22]. Meanwhile, the value for the liquid Ag in liquid Al was determined calorimetrically in two measurement series. The procedure employed during the measurement was analogous to that utilised in the determination of the enthalpy of formation, as described in the previous section. The necessary thermochemical data for pure elements for calculation were determined based on the relation in [25]. Table 4 outlines the determined values, accompanied by essential details concerning measurement parameters for each series.

Subsequently, measurements of the enthalpy of formation were conducted. The obtained results are summarised in Table 5, along with observed heat effects and the relevant information regarding the measurement parameters.

The average enthalpy of formation determined in measurement series A, based on five samples in an aluminium solvent at the temperature 1020 K, was equal to −14.4 kJ/mol∙at. Meanwhile, measurement series B conducted at 689 K in tin solvent for four samples exhibited an average enthalpy of formation value equal to −14.5 kJ/mol∙at. The obtained results from both measurements are consistent with each other. Based on those findings, it can be concluded that the solvent material does not influence the determined final value. The experimental values obtained are also consistent with the predictions presented in our previous work [17], in which the formation energy for *κ*-phase at 0 K was determined.

### 3.2. Theoretical Investigation

**Figure 5:**
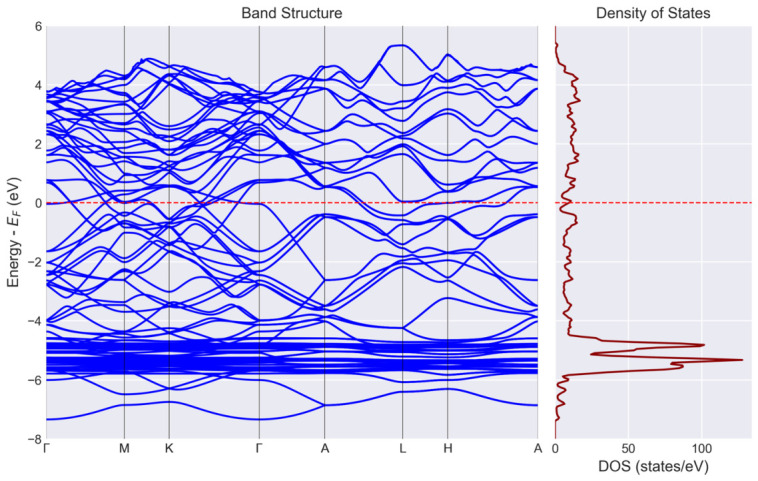
Calculated band structure (**left**) and density of states (**right**) of the *κ*-Ag_2_Mg_5_ phase.

Before the calculation of the electronic properties and defect formation energies, the structures of the Ag_2_Mg_5_, AgMg, AgMg_3_, Ag and Mg were relaxed. In this work, the electronic convergence threshold was set to 10^−6^ eV/atom, and the structural optimisation was performed until the Hellmann–Feynman forces on each atom were less than 0.001 eV/Å, corresponding to an EDIFFG value of −0.001. The calculations of electronic properties confirmed the metallic character of the *κ*-Ag_2_Mg_5_ phase. The calculated electronic structure, along with the density of states graph, is shown in Figure 5.

The band structure and density of states (DOS) for Ag_2_Mg_5_ show that it behaves as a metal. Multiple energy bands cross the Fermi level, and the DOS at the Fermi level is not zero, indicating that there are available electronic states for conduction. The complex bands near the Fermi level suggest contributions from both silver and magnesium atoms. Peaks in the DOS below the Fermi level likely correspond to filled silver d-states. The calculation was done using a self-consistent field (SCF) Fermi energy of 3.9586 eV. Overall, these results indicate that Ag_2_Mg_5_ should have good electrical conductivity.

After confirming the metallic character of the Ag_2_Mg phase, it became possible to calculate the defect formation energies. The issue of defect formation in the Ag_2_Mg_5_ phase is particularly interesting due to experimental observations [20], which revealed a slight non-stoichiometry of this phase. According to Castro et al. [20], the Wyckoff position 2c in the crystal lattice is occupied by 89% Ag and 11% Mg, resulting in a compositional shift toward Mg.

Since no other experimental studies have further investigated this phenomenon or examined the crystal structure in greater detail, it is worthwhile to apply a theoretical approach to explain this mixed occupancy. The Ag_2_Mg_5_ phase crystallises in the P6_3_/mmc space group, which allows for several types of point defects, including: Ag on an Mg site (Ag_Mg_), Mg on an Ag site (Mg_Ag_), Ag vacancies (V_Ag_), Mg vacancies (V_Mg_), interstitial Ag (i_Ag_) and interstitial Mg (i_Mg_).

Considering all possible defect types and their configurations within the crystallographic sites leads to 32 distinguishable defect scenarios that must be taken into account when evaluating formation energies. Before presenting the results of these calculations, it is necessary to clarify the role of chemical potentials. As shown in Equation (5), the chemical potential of atoms added to or removed from the crystal must be included in the calculation of defect formation energies.

Importantly, the Ag_2_Mg_5_ phase is not in equilibrium with pure Ag and Mg, but rather with competing intermetallic phases such as AgMg and AgMg_3_. Therefore, the chemical potentials must be constrained accordingly to ensure thermodynamic consistency. These constraints define the stability range of the phase and directly influence the calculated defect formation energies.

This leads to two limiting sets of chemical potentials:

Ag-rich limit (equilibrium with AgMg):

(5) $$\{\begin{array}{c} 2\mu_{\mathrm{Ag}}+5\mu_{\mathrm{Mg}}=E_{{\mathrm{Ag}}_{2}{\mathrm{Mg}}_{5}}^{\mathrm{fu}}\\ \mu_{\mathrm{Ag}}+\mu_{\mathrm{Mg}}=E_{\mathrm{AgMg}}^{\mathrm{fu}} \end{array}$$

Solving these equations yields:

(6) $$\mu_{\mathrm{Mg}}=\frac{E_{{\mathrm{Ag}}_{2}{\mathrm{Mg}}_{5}}^{\mathrm{fu}}-2E_{\mathrm{AgMg}}^{\mathrm{fu}}}{3},\mu_{\mathrm{Ag}}=E_{\mathrm{AgMg}}^{\mathrm{fu}}-\mu_{\mathrm{Mg}}$$

Mg-rich limit (equilibrium with AgMg_3_):

(7) $$\{\begin{array}{c} 2\mu_{\mathrm{Ag}}+5\mu_{\mathrm{Mg}}=E_{{\mathrm{Ag}}_{2}{\mathrm{Mg}}_{5}}^{\mathrm{fu}}\\ \mu_{\mathrm{Ag}}+3\mu_{\mathrm{Mg}}=E_{{\mathrm{AgMg}}_{3}}^{\mathrm{fu}} \end{array}$$

Solving these equations yields:

(8) $$\mu_{\mathrm{Mg}}=2E_{{\mathrm{AgMg}}_{3}}^{\mathrm{fu}}-E_{{\mathrm{Ag}}_{2}{\mathrm{Mg}}_{5}}^{\mathrm{fu}},\mu_{\mathrm{Ag}}=E_{{\mathrm{AgMg}}_{3}}^{\mathrm{fu}}-3\mu_{\mathrm{Mg}}$$

where $E_{{Ag}_{2}{Mg}_{5}}^{fu}=2\mu_{Ag}+5\mu_{Mg}$.

**Figure 6:**
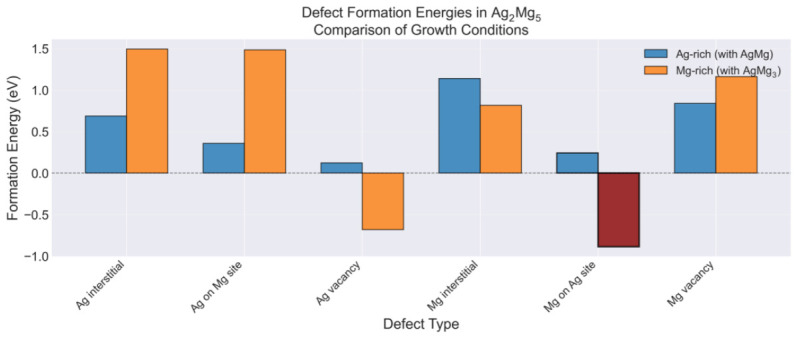
Formation energies of different types of defects in Ag-rich and Mg-rich growth conditions. The extra dark-red coloured orange bar indicates the most favourable defect in the *κ*-Ag_2_Mg_5_ phase.

After determining the chemical potentials of Ag and Mg under both Ag-rich and Mg-rich growth conditions, it was possible to calculate the defect formation energies. The results corresponding to the most energetically favourable defects are summarised in Table 6 and Figure 6.

The analysis of the results presented in Table 6 clearly indicates that the substitution of Ag by Mg (Mg_Ag_) is the most energetically favourable defect under Mg-rich growth conditions. Not only does this defect exhibit the lowest formation energy among all considered configurations, but its formation energy is also negative (−0.8855 eV), indicating that it is thermodynamically more stable than the pristine crystal lattice. This suggests a strong driving force for Mg atoms to occupy Ag lattice sites, thereby promoting the formation of antistites defects.

However, it should be noted that the extent of this substitution is limited. As Mg atoms progressively replace Ag atoms, the chemical potentials of the constituent elements are altered, which in turn affects the defect formation energies. Consequently, the concentration of such defects is self-limiting and must be determined under the constraints of thermodynamic equilibrium.

A graphical representation of the calculated formation energies under both Ag-rich and Mg-rich growth conditions is shown in Figure 6, which provides a comprehensive overview of defect stability across different growth environments. This visualisation highlights the strong dependence of defect energetics on the value of chemical potential conditions and further emphasises the dominant role of Mg_Ag_ defects in Mg-rich regimes.

The next most energetically favourable defect is the Ag vacancy (V_Ag_), also under Mg-rich growth conditions. The formation of Ag vacancies similarly contributes to a shift in the overall composition toward Mg-rich stoichiometry. However, experimental studies by Castro et al. [20] did not report the presence of vacancies in the Ag_2_Mg_5_ crystal structure, suggesting that their concentration may be low or below the detection limit of the employed characterisation techniques.

All other vacancy types, as well as interstitial defects, exhibit positive formation energies under both Ag-rich and Mg-rich growth conditions. This indicates that their formation is energetically unfavourable, and thus their concentrations are expected to be negligible in equilibrium. As a result, these defects are likely to play a significant role in determining the structural or compositional properties of the Ag_2_Mg_5_ phase.

## 4. Conclusions

This work is a continuation of research on the thermodynamic properties of the Ag-Mg system. The findings from the experimental and theoretical investigation presented in this work offer an interesting insight into the properties of the *κ*-Ag_2_Mg_5_ phase and expand on the existing knowledge of this phase in extant literature. Based on the obtained results, the following conclusions can be drawn:

1. The XRD analysis confirmed the presence of the *κ*-phase as a dormant phase in the alloy produced in this study.
2. The results of the enthalpy of formation obtained from the calorimetric measurement for this compound in an Al solvent and a Sn solvent were equal, respectively, −14.4 ± 0.32 kJ/mol∙at and −14.5 ± 0.42 kJ/mol∙at.
3. No significant effect of the solvent material on the standard enthalpy of formation was observed.
4. The ab initio results are in excellent agreement with the experimental observations reported by Castro et al. [20], explaining the non-stoichiometry of the Ag_2_Mg_5_ phase through antisite substitution (Mg_Ag_) as the dominant defect mechanism. This strong theory–experiment consistency underscores the reliability of the computational approach and provides key insight into the defect chemistry and stability of the system.

## Figures and Tables

**Table 1:** Detailed information on all materials applied.

Chemical Name	Source	Purity ^1^
Silver	Innovator Sp. Z.o.o, Gliwice, Poland	99.99
Magnesium	Goodfellow Cambridge Ltd., Huntington, England	99.99
Argon	Pioniergas, Krakow, Poland	99.9999
Tin	Alfa Aesar, Thermo Scientific Kandel GmBH, Kandel, Germany	99.999
Aluminium	Alfa Aesar, Thermo Scientific Kandel GmBH, Kandel, Germany	99.99

^1^ Certified purity in mass %.

**Table 2:** Average local chemical composition of the alloy before and after homogenisation.

EDS	Atomic %	
	Ag	Mg
Before homogenisation (Figure 1a)	26.8 (±1.2)	73.2 (±0.8)
After homogenisation (Figure 1b)	27.5 (±1.3)	72.5 (±0.7)

**Table 3:** Detailed local chemical composition of the alloy after homogenisation.

EDS	Atomic %	
	Ag	Mg
Area A (Figure 1b)	27.0 (±1.2)	73.0 (±0.8)
Area B (Figure 1b)	44.7 (±1.6)	55.3 (±0.4)

**Table 4:** Determined values of the limiting partial enthalpy of solution of liquid Ag in Al.

Measurements Parameters	Sample No.	Mass of the Dropped Sample [g]	At. % of Ag in Al Solvent	Heat Effect ${\Delta H}^{\mathrm{ef}}$ [kJ/mol]	Limiting Partial $\mathrm{Enthalpy}\;\mathrm{of}\;\mathrm{Solution}\;\Delta_{\mathrm{sol}}{\bar{H}}_{\mathrm{Ag}\left(l\right)}^{\infty }$ [kJ/mol]
**Series I** Calibration constant: *K* = 0.00000712 kJ/μVs *u*(*K*) = 0.000000034 kJ/μVs Enthalpy of pure elements: ${\Delta H}_{\mathrm{Ag}}^{T_{D}\to T_{M}}$ = 33.1948 kJ/mol ${\Delta H}_{\mathrm{Al}}^{T_{D}\to T_{M}}$ = 34.0672 kJ/mol Temperatures: *T*_D_ = 298 K (±1 K) *T*_M_ = 1098 K (±1 K)	1	0.1123	0.42	40.4	7.2
	2	0.1099	0.63	40.2	7.0
	3	0.1060	0.84	40.0	6.8
	4	0.1133	1.25	40.8	7.6
**Series II** Calibration constant: *K* = 0.00000775 kJ/μVs *u*(*K*) = 0.00000015 kJ/μVs Enthalpy of pure elements: ${\Delta H}_{\mathrm{Ag}}^{T_{D}\to T_{M}}$ = 33.1948 kJ/mol ${\Delta H}_{\mathrm{Al}}^{T_{D}\to T_{M}}$ = 34.0672 kJ/mol Temperatures: *T*_D_ = 298 K (±1 K) *T*_M_ = 1098 K (±1 K)	1	0.1107	0.22	40.2	7.0
	2	0.1550	0.51	40.3	7.2
	3	0.1351	0.77	38.8	5.6
	4	0.0922	0.95	41.3	8.1
	5	0.1061	1.15	40.3	7.1
**Average ^1^**				**40.3**	**7.1**
**Standard deviation ^1^**				**0.7**	**0.7**

^1^ Calculated based on both measurement series.

**Table 5:** Heat effects of solution and the standard enthalpies of formation.

Measurements Parameters	Solvent and Calibration Material	Sample No.	Heat Effects ${\Delta H}_{x_{\mathrm{Ag}}x_{\mathrm{Mg}}}^{0}$	Enthalpy of Formation Δ_f_*H* [kJ/mol∙at.]
**Series A** Calibration constant: *K* = 0.00000837 kJ/μVs *u*(*K*) = 0.00000009 kJ/μVs Enthalpy of pure elements: ${\Delta H}_{\mathrm{Ag}}^{T_{D}\to T_{M}}$ = 30.9246 kJ/mol ${\Delta H}_{\mathrm{Mg}}^{T_{D}\to T_{M}}$ = 29.5837 kJ/mol ${\Delta H}_{\mathrm{Al}}^{T_{D}\to T_{M}}$ = 31.6151 kJ/mol Limiting partial enthalpy of solution in Al: $\Delta_{\mathrm{sol}}{\bar{H}}_{\mathrm{Ag}\left(l\right)}^{\infty }$ = 7.1 kJ/mol $\Delta_{\mathrm{sol}}{\bar{H}}_{\mathrm{Mg}\left(l\right)}^{\infty }$ = −8.6 kJ/mol [22] *T*_D_ = 297 K (±1 K) *T*_M_ = 1020 K (±1 K)	Al	1	40.1	−14.2
		2	39.8	−13.9
		3	40.6	−14.8
		4	40.3	−14.4
		5	40.3	−14.8
		**Average**	**40.2**	**−14.4**
		**Standard deviation**	**0.32**	**0.32**
**Series B** Calibration constant: *K* = 0.0000138 kJ/μVs *u*(*K*) = 0.0000001 kJ/μVs Enthalpy of pure elements: ${\Delta H}_{\mathrm{Ag}}^{T_{D}\to T_{M}}$ = 21.3300 kJ/mol ${\Delta H}_{\mathrm{Mg}}^{T_{D}\to T_{M}}$ = 18.2026 kJ/mol ${\Delta H}_{\mathrm{Sn}}^{T_{D}\to T_{M}}$ = 18.3215 kJ/mol Limiting partial enthalpy of solution in Sn: $\Delta_{\mathrm{sol}}{\bar{H}}_{\mathrm{Ag}\left(l\right)}^{\infty }$ = 3.5 kJ/mol [23] $\Delta_{\mathrm{sol}}{\bar{H}}_{\mathrm{Mg}\left(l\right)}^{\infty }$ = −33 kJ/mol [24] Temperatures: *T*_D_ = 298 (±1 K) *T*_M_ = 689 (±1 K)	Sn	1	10.4	−13.9
		2	11.2	−12.5
		3	11.3	−14.6
		4	11.3	−14.8
		**Average**	**11.0**	**−14.5**
		**Standard deviation**	**0.42**	**0.42**

**Table 6:** The most energetically favourable defects in the *κ*-Ag_2_Mg_5_ phase.

Defect Type	Formation Energy [eV]	
	Ag-Rich	Mg-Rich
i_Ag_	0.6883	1.4939
Ag_Mg_	0.3571	1.4849
V_Ag_	0.1238	−0.6818
i_Mg_	1.1385	0.8162
Mg_Ag_	0.2423	−0.8855
V_Mg_	0.8406	1.1629

## Data Availability

The original contributions presented in this study are included in the article. Further inquiries can be directed to the corresponding author.
